# Efficacy of mesenchymal stem cell therapy in rodent models of radiation-induced xerostomia and oral mucositis: a systematic review

**DOI:** 10.1186/s13287-023-03301-y

**Published:** 2023-04-12

**Authors:** Zirui Guan, Jiaxin Zhang, Nan Jiang, Mingyan Tian, Hongyong Wang, Bing Liang

**Affiliations:** 1 The Second Hospital of Jilin University, Changchun City, 130022 Jilin Province People’s Republic of China; 2 School of Nursing, Jilin University, Changchun City, 130021 Jilin Province People’s Republic of China

**Keywords:** Mesenchymal stem cells, Xerostomia, Oral mucositis, Ionizing radiation, Systematic review

## Abstract

**Background:**

Radiation-induced xerostomia and oral mucositis are serious complications of radiation therapy for head and neck cancers. Current treatment options have limited efficacy. Mesenchymal stem cell (MSC) therapy has shown promising results in supporting the restoration of glandular secretion function and the regeneration of damaged tissues. This study aim to (1) assess the quality of evidence for MSCs treatment in rodent models of radiation-induced oral complications and (2) determine whether MSCs can improve the therapeutic effect of radiation-induced oral mucositis.

**Methods:**

Intervention studies using MSCs in rodent models were comprehensively retrieved in the Pubmed, Medline, Embase, Web of Science, and Cochrane library databases on June 1, 2022. The quality of all in vivo experiments was assessed using SYRCLE, and this article is written following the PRISMA guidelines.

**Results:**

A total of 12 studies were included in this systematic review. The study found that in animal models of radiation-induced xerostomia, MSCs could increase salivary protein secretion, improve SFR, shorten the salivary lag time, anti-apoptosis, etc. In animal models of radiation-induced oral mucositis, MSCs improve the micromorphology and macromorphology of RIOM. Moreover, the effect of MSCs on the modification of ulcer duration and latency may be related to the time of MSCs transplantation but further studies are needed.

**Conclusion:**

The results of our systematic review suggest that MSCs appeared to be effective in the treatment of radiation-induced xerostomia and oral mucositis.

**Graphical Abstract:**

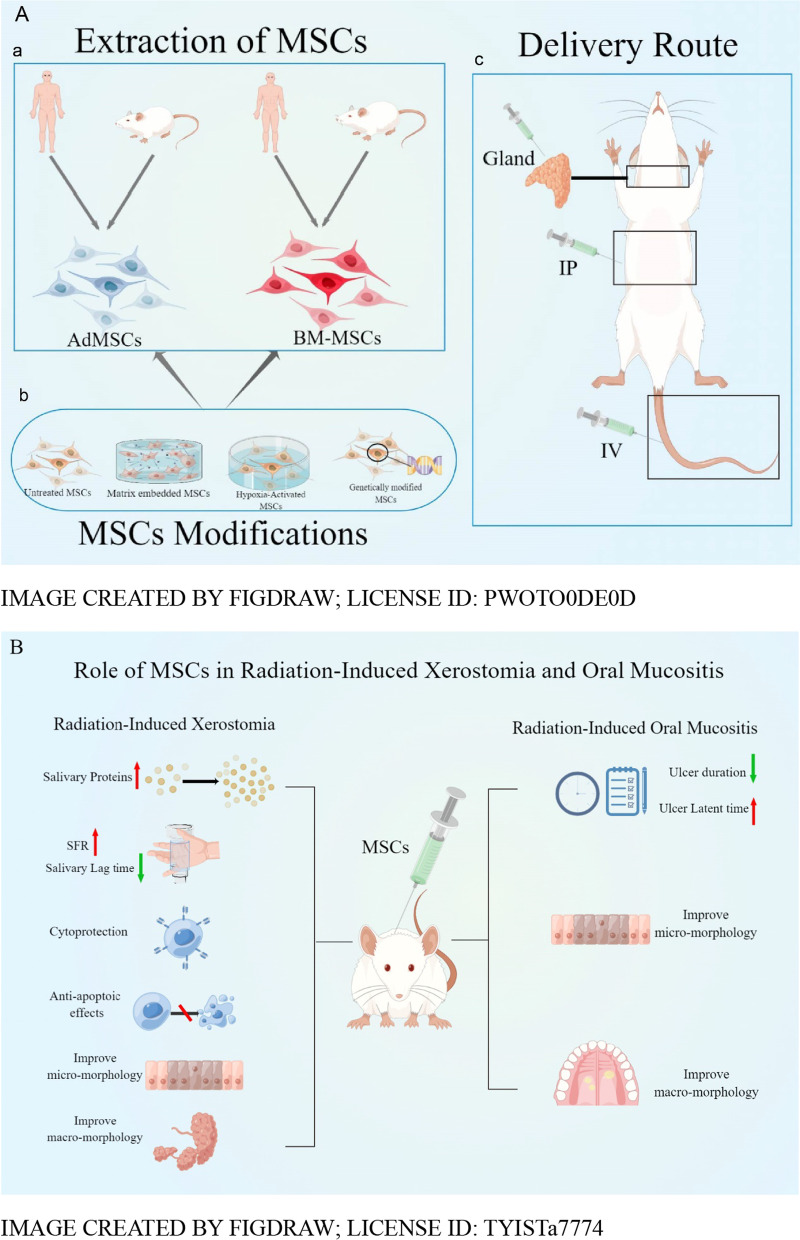

**Supplementary Information:**

The online version contains supplementary material available at 10.1186/s13287-023-03301-y.

## Introduction

Ionizing radiation used as external radiotherapy, brachytherapy, and targeted radionuclide therapy plays a pivotal role in the treatment of patients with head and neck cancer (HNC). Approximately 80% (range 73.9–84.4%) of all HNC patients receive radiotherapy at least once during their disease [1]. Despite the advantages of radiotherapy in preserving tissue architecture, and the modern radiotherapy techniques that have been developed using advanced, highly conformal RT (e.g. IMRT) delivery methods. It is not always possible to completely avoid toxicity [2]. No technique can completely protect normal tissue from radiation. So patients will always experience some degree of radiation-related toxicity [3]. Many patients with head and neck cancer receive high-dose radiotherapy to large areas of the mouth, maxilla, mandible, and salivary glands [4], causing many acute and long-term oral adverse effects, such as mucositis, xerostomia, taste disturbances, vascular damage, increased risk of dental caries and periodontal disease, and the most severe radiation-induced osteonecrosis [5]. Oral complications due to radiotherapy can lead to high morbidity and reduced quality of life, increasing the cost of treatment and management. Among these, severe hypofunction of the salivary glands and early radiation reactions to the oral mucosa is particularly common and serious adverse effects of radiotherapy for advanced head and neck tumors [6]. The prevalence of oral dryness after radiotherapy for head and neck cancers ranges from 74 to 85% [7], and the prevalence of radiation-induced oral mucositis (RIOM) in head and neck radiotherapy approaches 100% [8, 9]. RIOM is a radiotherapy-induced condition affecting the inflammation of the oral mucosa, characterized by erythema, a painful ulcerative lesion affecting the oral lining. Due to oral mucosal damage, patients complain about burning sensations, oral pain, and ulcers, which lead to increased pain whenever the patients try to eat or drink [4], requiring the use of pain medication during treatment. In addition, there may even be bleeding, dysphagia, and dysarthria, which in turn interfere with the radiation treatment process and alter the local control of the tumor and ultimately affect the patient's survival. Radiation-induced xerostomia is a combination of irradiation-induced changes in salivary gland function or saliva quantification and quality. It combines subjective complaints of dry mouth and objective reduction in saliva production. Patients present with difficulty chewing and swallowing dry food, impaired vocalization, persistent dryness and burning sensation in the mouth, and tasting disturbances, which can be severe and may result in loss of taste, loss of appetite, and weight loss [10–12]. This can be very distressing for the patients and even hinder treatment or prompt abandonment. The two radiation-induced oral complications, oral mucositis, and xerostomia present formidable challenges to healthcare providers. Safer and more effective strategies need to be found to reduce their severity and deleterious effects on basic life functions and quality of life. Several recent studies suggest that mesenchymal stem cell therapy may be a viable treatment option for radiation oral mucositis and xerostomia [13–15].

Mesenchymal stem cells are cells that originate from the mesoderm of embryonic development and are widely found in a variety of tissues such as the umbilical cord, bone marrow, and adipose and have a strong capacity for self-renewal and differentiation [16] and have been used to treat various diseases such as neurodegenerative diseases [17, 18], spinal cord injuries [19], hematologic disorders [20], graft-versus-host disease GVHD [21, 22], and reproductive system disorders [23]. Special attention is paid to the fact that in recent years, MSCs have been widely used in various inflammatory diseases and salivary gland injury diseases. Since MSCs have functions such as directed differentiation [24], regulation of immunity [25], antioxidant [26], anti-apoptosis [27], promotion of cell regeneration [28], and angiogenesis [29], they have good therapeutic effects in various inflammatory diseases and salivary gland injury diseases.

These findings have led researchers to further think about the possibility of applying MSCs in the treatment of radiation injury to solve the key and difficult problems of the oral gland and mucosa repair after radiation injury. Jae-Yol Lim et al. have proposed that bone marrow-derived clonal mesenchymal stem cells (BM-MSCs) can differentiate into salivary epithelial cells and preserve salivary gland function through transplantation and transdifferentiation to improve salivary injury after radiation. BM-MSCs also can be used as a cell-based therapeutic source to restore radiation-induced hyposalivation [30]. Lee et al. have shown that adipose-derived mesenchymal stem cells (AdMSCs) secretion can reduce tissue damage by inhibiting apoptosis, inflammation, and fibrotic remodeling through a variety of trophic factors with various properties produced by paracrine activity. And AdMSCs promote angiogenesis and endogenous stem cells recruitment to repair and remodel salivary gland (SG) structures [31]. AdMSCs synthesize and release a variety of paracrine factors that can also contribute to mucosal damage by enhancing cell proliferation or inhibiting epithelial cell apoptosis, or by a combination of both to promote mucosal repair [18, 32–34]. The implanted MSCs also downregulate pro-inflammatory cytokines and significantly reduce cellular reactive oxygen species (ROS) levels to relieve oral mucositis [35].

Although MSC-based approaches have proven beneficial in models of xerostomia and oral mucositis after radiation injury, the optimal protocol, manipulation, or cellular product remains controversial. Therefore, the purpose of this review is to evaluate the efficacy and safety of MSCs in preclinical studies for radiation-induced xerostomia and oral mucositis and to assess the potential use of MSCs in future human trials.

## Method and materials

### Search strategy

Five databases (PubMed, Embase, the Web of Science, the Cochrane Library, and Medline) were searched from their inception dates to June 1st, 2022, The queries and study strings applied using Boolean operators are listed in Additional file 1. Database queries were performed on two separate topics, and differences were resolved after discussions with a third party. All citations were managed using Zotero software. In addition, the reference lists of all publications included in this review were hand-searched for additional studies. Retrieval strategies, screening, and data selection are carried out following PRISMA standards [36]. The review was registered in the International Prospective Register of Systematic Reviews (PROSPERO) under the registration number CRD42022299487.

### Inclusion and exclusion criteria

Inclusion criteria for the screened publications were (1) studies involving animal models of radiation-induced oral disease (all species and sexes); (2) all animal models of radiation-induced oral disease were treated with MSCs; (3) received any delivery route and included multiple dosing regimens; (4) include efficacy outcome studies; (5) studies have a control group and the control group is a placebo control using PBS. Applied exclusion criteria were (1) all inclusion criteria were not fulfilled; (2) meeting abstracts, case reports, and case series; (3) reviews or meta-analysis; (4) the study was duplicated; (5) studies published in a non-English language.

### Study selection

After obtaining the results of the electronic database search, the two authors first examined the titles, abstracts, and full texts of the studies separately. For eligible articles, which would be read further in full to clarify whether to include in the final study, the two authors negotiated divergentially at any stage of review and consulted third parties if necessary. Each original study was included only once, and we excluded studies without raw data.

### Data extraction

We used Excel software to create a table to extract information. Two authors independently extracted key information from each study. The extracted information in the table includes first author, year of publication, study country, study type, type of ionizing radiation, disease model, experimental subjects, type of MSCs, MSCs implantation method, dose, follow-up time, and outcome indicators. If data were missing, the author of the article would be contacted by email for specific data information. If the author did not reply to the message, a second email contact was sent. If there was still no reply, the data were considered unavailable.

### Quality assessment

Two authors independently assessed the methodological quality of the included studies. A third author was required in case of any dispute during data extraction and quality assessment. The quality of all in vivo experiments was assessed using the Risk of Bias in Animal Studies tool of the Systematic Review Centre for Laboratory Animal Experimentation (SYRCLE) [37]. The quality of the studies was judged as “high risk,” “low risk,” or “unclear.” The SYRCLE Risk of Bias tool for animal studies has 10 items: They fall into 6 areas, including selection bias (Random Sequence Generation, Baseline Characterization, Allocation Concealment), performance bias (Randomized Housing, Blinding), detection bias (Randomized Outcome Assessment, Blinding), attrition bias (Incomplete Outcome Data), reporting bias (Selective Outcome Reporting) and other (Other Sources of Bias).

## Results

### Study selection

**Fig. 1:**
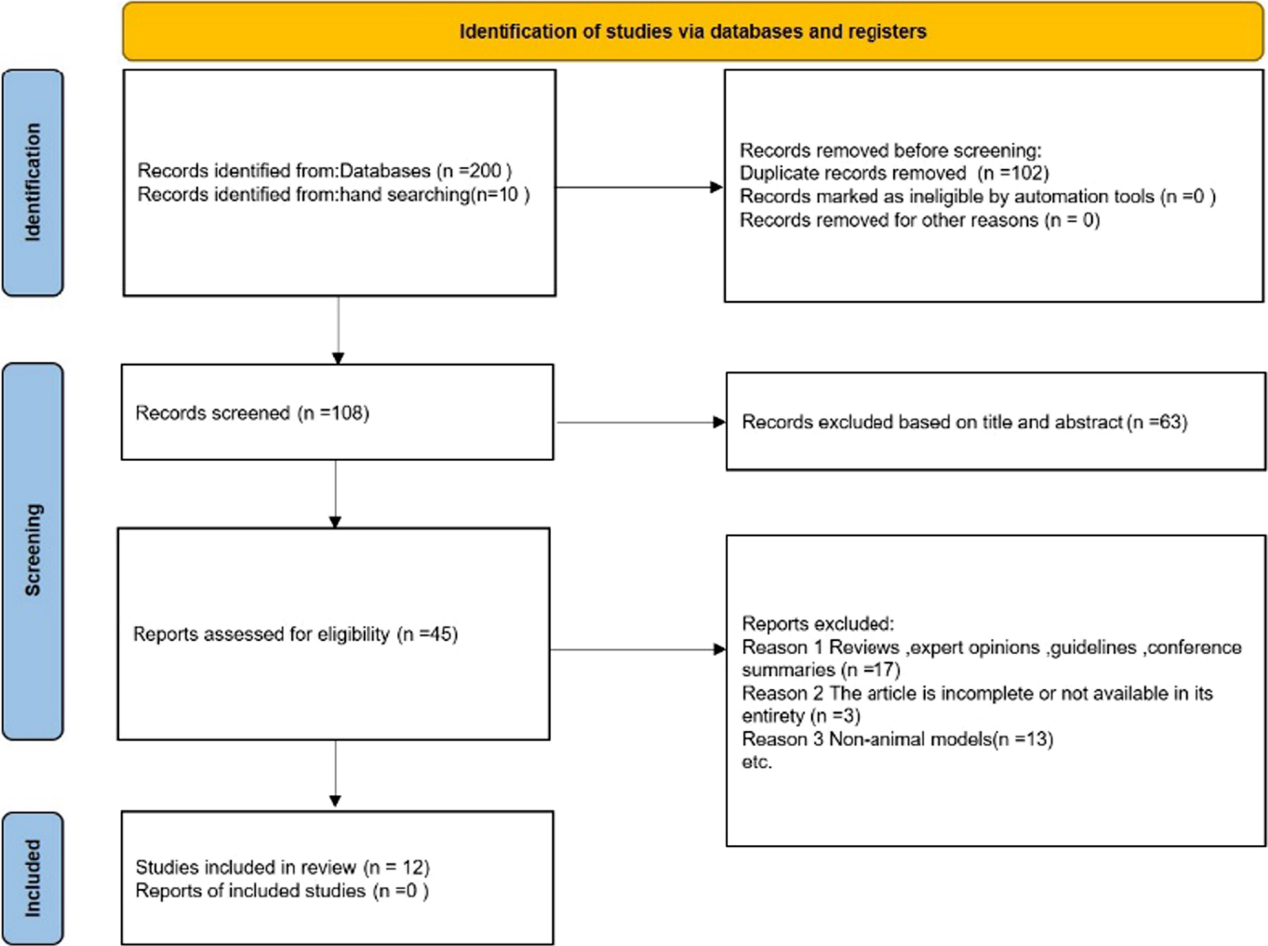
Flow diagram of the study selection

Based on the search strategy, we included 200 studies related to MSCs for the treatment of radiation and oral mucositis, and 102 duplicate articles were removed using Zotero software. Sixty-three articles were removed by reading the titles and abstracts, and the remaining 45 were reviewed in full text. Seventeen reviews, expert opinions, guidelines, or conference summaries were excluded. Because articles were incomplete or not available in their entirety, 3 articles were excluded. Furthermore, 13 articles were not included because of non-animal models. Finally, after study selection, 12 articles were included in this systematic review (Fig. 1) (If multiple interventions were offered in a single study, each intervention was considered independent).

### Study characteristics

**Fig. 2:**
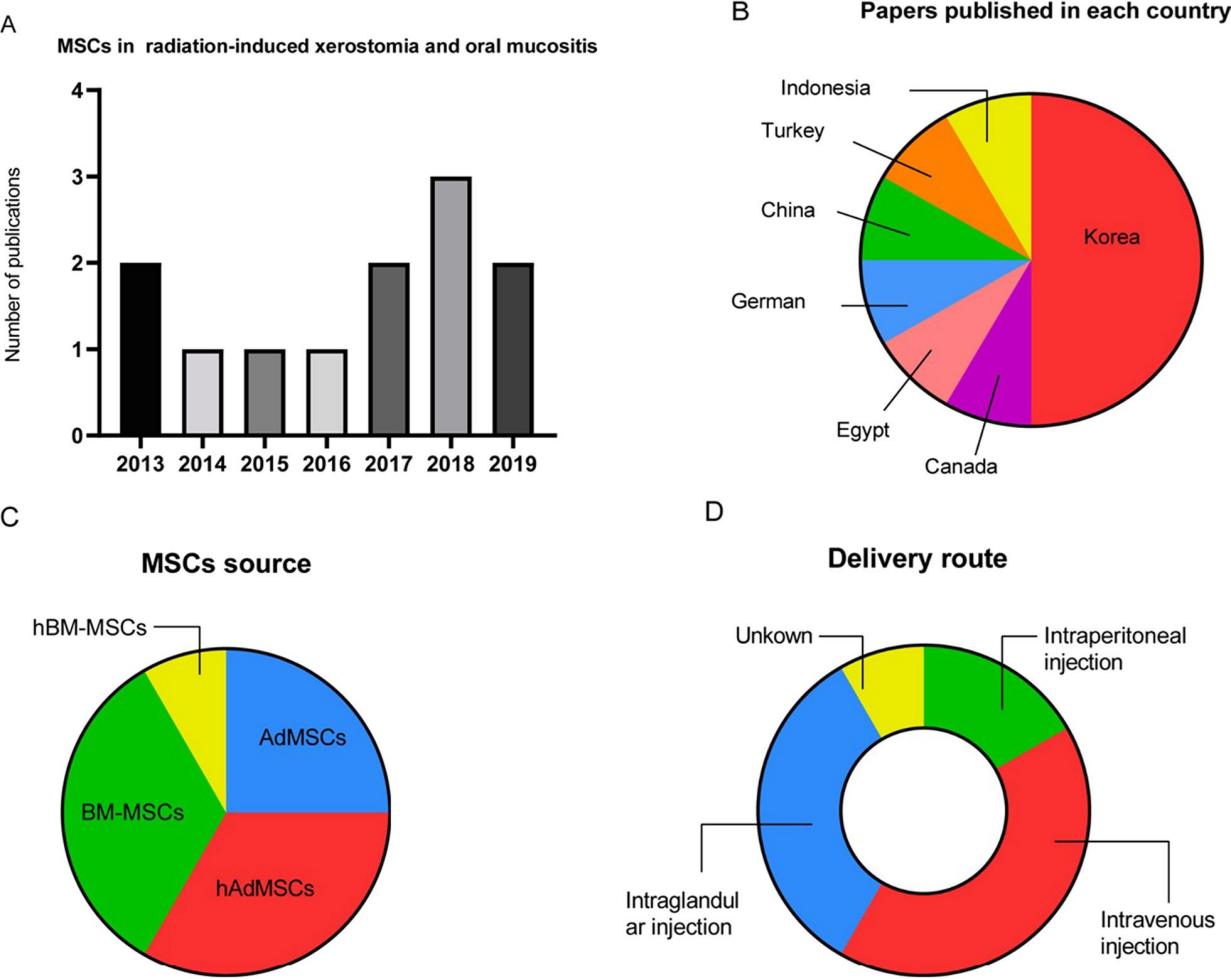
Overall characteristics of the 12 studies. **A** Number of publications per year. **B** Number of papers published per country. **C** MSC source. **E** Route of delivery

The basic characteristics of twelve articles are listed in Tables 1, 2, and Fig. 2 [13–15, 30, 35, 38–44]. The articles were published between 2013 and 2019. The studies conducted in different countries including Korea (*n* = 6) [15, 30, 39–42], Canada(*n* = 1) [38], Egypt (*n* = 1) [13],German(*n* = 1) [14], China(n = 1) [35],Turkey(*n* = 1) [43] and Indonesia(*n* = 1) [44]. The studies were conducted in rodents (rats [13, 43, 44] and mice [14, 15, 30, 38–40, 42, 45]). The animal models in all studies were induced by radiation. Stem cell types included BM-MSCs (*n* = 5) [13, 14, 30, 35, 44] and AdMSCs (*n* = 7) [15, 38–43]. The doses of interventions ranged from 10^5^ to 10^7^. MSCs were injected into a vein in five animal studies [13, 14, 35, 39, 42], injected into the abdominal cavity in two studies [38, 43], and injected into a gland in the rest of five studies [15, 30, 40, 41, 44]. The timing of cell administration ranged from 0 h to 28 days after induction of the model. The duration of the follow-up period ranged from 7 days to 6 months. For the outcome indicators involved in the study, we also summarized and concluded in Tables 3 and 4. Therefore, the systematic review included a total of twelve animal studies involving 659 animals.

**Table 1:** Characteristics of animal studies

Author/years	Country	Study designs	Species	Strain	Sex	Total number of animals	IR Types	Irradiation dose	Follow-up time
*Radiation-induced xerostomia*									
An et al. 2015 [39]	Korea	RCT	Mice	C3H	Female	105	X-ray	15 Gy	16W
Kim et al. 2019 [40]	Korea	RCT	Mice	C57BL/6	Female	45	Radioiodine	0.01 mCi/g	16W
Choi et al. 2018 [15]	Korea	RCT	Mice	C3H	Female	140	X-ray	15 Gy	16W
Shin et al. 2018 [41]	Korea	RCT	Mice	C3H	Female	12	X-ray	15 Gy	12W
Lim,et al. 2013 [30]	Korea	RCT	Mice	C57BL/6	Unkown	24	X-ray	15 Gy	12W
Saylam et al. 2017 [43]	Turkey	RCT	Rats	Wistar albino	Female	60	Radioiodine	2 mCi	6 M
Lim et al. 2013 [42]	Korea	RCT	Mice	C3H	Female	60	X-ray	15 Gy	12W
Mulyani et al. 2019 [44]	Indonesia	RCT	Rats	Wistar	Male	40	X-ray	15 Gy	4W
*Radiation-induced oral mucositis*									
Maria et al. 2016 [38]	Canada	RCT	Mice	BALB/c	Male	45	X-ray	18 Gy	21D
Elsaadany et.al.2017 [13]	Egypt	RCT	Rats	Albino	Male	60	X-ray	13 Gy	7D
Schmidt et al. 2014 [14]	German	RCT	Mice	C3H/Neu	Male	50	X-ray	15 Gy	3W
Shen et al. 2018 [35]	China	RCT	Mice	C57	Male	18	X-ray	16 Gy	10D

*D* Days; *M* Months, *RCT* Randomized controlled trial, *W* Weeks

**Table 2:** Mesenchymal stem cell characteristics

Author/Years	Sources	Control group	Manipulation	Dose	Volume	Delivery route
*Radiation-induced xerostomia*						
An et al. 2015 [39]	hAdMSCs	PBS	Hypoxia	1 × 105	500μl	Intravenous injection
Kim et al. 2019 [40]	hAdMSCs	PBS	Non	1 × 105	Unkown	Intraglandular injection
Choi et al. 2018 [15]	hAdMSCs	PBS	Matrix	1 × 105	20 μl	Intraglandular injection
Shin et al. 2018 [41]	hAdMSCs	PBS	Hypoxia	2 × 105	10 μl	Intraglandular injection
Lim et al. 2013 [30]	BM-MSCs	PBS	Non	1 × 105	15 μl	Intraglandular injection
Saylam et al. 2017 [43]	AdMSCs	PBS	Non	2 × 106	Unkown	Intraperitoneal injection
Lim et al. 2013 [42]	AdMSCs	PBS	Non	1 × 106	Unkown	Intravenous injection
Mulyani et al. 2019 [44]	BM-MSCs	PBS	Hypoxia	Unkown	Unkown	Unkown
*Radiation-induced oral mucositis*						
Maria et al. 2016 [38]	AdMSCs	PBS	Non	2.5 × 106	500 μl	Intraperitoneal injection
Elsaadany et al. 2017 [13]	BM-MSCs	PBS	Non	1 × 107	0.2 ml	Intravenous injection
Schmidt et al. 2014 [14]	BM-MSCs	PBS	Non	6 × 106	Unkown	Intravenous injection
Shen et al. 2018 [35]	hBM-MSCs	PBS	Genetic	Unknown	Unknown	Intravenous injection

*AdMSCs* Adipose tissue-derived mesenchymal stem cells; *BM-MSCs* Bone marrow-derived mesenchymal stem cells; *PBS* Phosphate buffered saline

**Table 3:** Evaluation of the efficacy of mesenchymal stem cells in the treatment of radiation-induced Xerostomia

Author/years	Salivary proteins	Salivary gland flow rate	Salivary lag time	Cytoprotection	Anti-apoptotic effects	Micromorphology	Macromorphology
An et al. 2015 [39]	+	+	+	+	+	+	+
Kim et al. 2019 [40]	+	+	+	+	+	+	+
Choi et al. 2018 [15]	+	+	+	+	+	+	+
Shin et al. 2018 [41]	+	+	+	+	–	+	+
Lim et al. 2013 [30]	+	+	+	–	+	+	+
Saylam et al. 2017 [43]	–	–	–	–	–	–	–
Lim et al. 2013 [42]	+	+	+	–	+	+	+
Mulyani et al. 2019 [44]	+	–	–	–	+	+	–

Mention: +; Not Mention: –

**Table 4:** Evaluation of the efficacy of mesenchymal stem cells in the treatment of radiation-induced Oral mucositis

Author/years	Ulcer latency time	Duration of ulcers	Micromorphology	Macrosmorphology	The side effects of RIOM	BCL-2	The tolerance of the oral mucosa	Pro-inflammatory cytokines
Maria et al. 2016 [38]	+	+	+	+	+	–	–	–
Elsaadany et al. 2017 [13]	–	–	+	–	–	+	–	–
Schmidtet al. 2014 [14]	+	+	–	–	–	–	+	–
Shen et al. 2018 [35]	–	–	+	+	–	–	–	+

Mention: + ; Not Mention: –

### Risk of bias

**Fig. 3:**
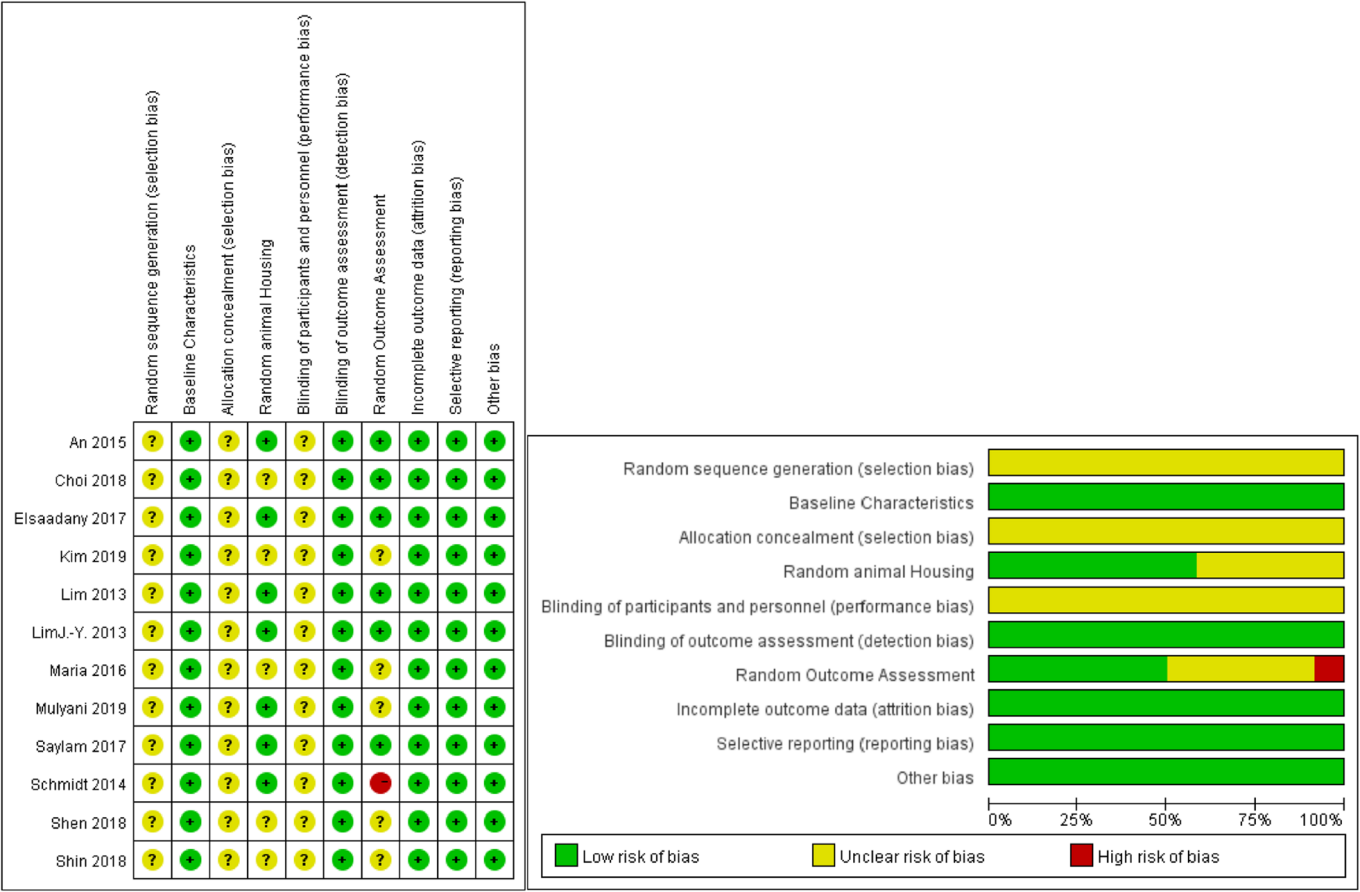
SYRCLE’s RoB tool for each experimental animal study

Table 5 and Fig. 3 provide a detailed assessment of the quality of each study. All studies had selection bias secondary to baseline characteristics, detection bias secondary to randomized outcome assessment, attrition bias, reporting bias, and other biases [13–15, 30, 35, 38–44]. Among them, blinding is the main weakness of the study design. We think this may be due to the fact that the implementation of blinding in animal experiments is usually not feasible.

**Table 5:** SYRCLE’s RoB tool for each experimental animal study

	Random Sequence Generation	Baseline Characteristics	Allocation Concealment	Random Housing	Blinding (study Team)	Random Outcome Assessment	Blinding (Outcome Assessors)	Incomplete Outcome Data	Selective Outcome Reporting	Other sources of bias
	Selection bias			Performance bias		Detection bias		Attrition bias	Reporting bias	Other
An	?	+	?	+	?	+	+	+	+	+
Maria	?	+	?	?	?	+	?	+	+	+
Kim	?	+	?	?	?	+	?	+	+	+
Elsaadany	?	+	?	+	?	+	+	+	+	+
Schmidt	?	+	?	+	?	+	–	+	+	+
Choi	?	+	?	?	?	+	+	+	+	+
Shen	?	+	?	?	?	+	?	+	+	+
Shin	?	+	?	?	?	+	?	+	+	+
Lim	?	+	?	+	?	+	+	+	+	+
Saylam	?	+	?	+	?	+	+	+	+	+
Lim J.-Y	?	+	?	+	?	+	+	+	+	+
Mulyani	?	+	?	+	?	+	?	+	+	+

### Radiation-induced xerostomia

#### Salivary proteins

A total of seven studies [15, 30, 39–42, 44] reported the secretion of salivary proteins (salivary amylase, epidermal growth factor, mucin) after MSCs treatment. Four of them [15, 39–41] reported changes in salivary amylase and epidermal growth factor (EGF), with significantly higher levels of salivary amylase expression after MSCs infusion relative to control and mean EGF levels also significantly increased relative to the control group. Two studies [30, 42] reported changes in salivary amylase and mucin. The group treated with MSCs showed more amylase and mucin content in the tissue compared to the control group. One study [44] reported changes in salivary amylase only. Compared to the control group, the experimental group increased amylase activity after the use of MSCs and the difference was statistically significant (*p* < 0.05). Also, Mulyani et al. [44] found that MSCs activated under hypoxic conditions resulted in a further increase in amylase activity secreted by salivary glands compared to MSCs under normoxic conditions (*p* < 0.05).

#### Salivary gland flow rate and lag time

A total of six studies [15, 30, 39–42] recorded changes in salivary gland flow rate (SFR) and salivary lag time. SFR was calculated as the ratio of post-IR SFR to pre-IR SFR (Mean ± SEM). Salivary lag time was also calculated as the ratio of post-IR salivary lag time to pre-IR salivary lag time (Mean ± SEM). The six studies did not choose the same follow-up time. Three studies [15, 39, 40] chose to measure SFR and salivary lag time at 16 weeks after irradiation. And three studies [30, 41, 42] chose to measure SFR and salivary lag time at 12 weeks after irradiation. These six studies came to the consistent conclusion that the mean SFR was significantly higher in the MSCs group than in the control group, and the mean salivary lag time in the MSCs group time was significantly lower than that of the control group.

#### Cytoprotection

Four studies [15, 39–41] examined immunohistochemical markers to investigate the cytoprotective effects of MSCs on salivary epithelial, endothelial, myoepithelial, and progenitor cells. Two studies [15, 39] performed immunohistochemistry for CD31 (endothelial cells), AQP5 (a marker of salivary epithelial cells), α-SMA (myoepithelial cells), and c-Kit (progenitor cells) and showed that the MSCs group represented a significantly higher expression of CD31, AQP5, α-SMA, and c-Kit than the control group (*p* < 0.05). Kim et al. [40] also discussed the changes in AQP-5, CD31, and the marker AQP-5, CD31 were significantly increased in the treated group compared to the control group (*p* <  < 0.05). The study by Shin et al. [41] similarly reported that compared to the control, the MSCs group with enhanced expression of salivary epithelial cell markers (AMY1A, AQP5, KRT7, and KRT18).

#### Anti-apoptotic effects

Six studies [15, 30, 39, 40, 42, 44] reported the anti-apoptotic effect of MSCs on salivary gland cells. Five studies [15, 30, 39, 40, 42] performed terminal dUTP node end labeling (TUNEL) to determine the number of apoptotic cells in the MSCs and control groups. These studies showed that the number of TUNEL-positive apoptotic cells was significantly lower in the MSCs group than in the control group. One of the studies by An et al. [39] also confirmed that the MSCs group under hypoxic conditions led to an increased anti-apoptotic effect of cells compared with the MSCs group under normoxic conditions (*p* < 0.01 at 1 or 2 weeks after treatment and *p* < 0.001 at 4 weeks after treatment). The expression of BCL-2, an anti-apoptotic protein secreted by cells, was upregulated and indicated a decrease in apoptotic activity. The study of Mulyani et al. [44] of BCL-2 expression was measured in the MSCs group and control group. The results showed that the expression of Bcl-2 was significantly stronger in the MSCs group than in the control group. In addition, Bcl-2 expression was stronger under hypoxic conditions compared to MSCs under normoxic conditions.

#### Micromorphology

Seven studies [15, 30, 39–42, 44] evaluated the improvement of micromorphology after treatment with MSCs. Three studies [15, 39, 40] found that 16 weeks after IR, the structure, fibrosis density, and degree of inflammatory cell infiltration of salivary gland ducts and periducts were well protected in rats or mice treated with MSCs compared to controls. Three studies [30, 41, 42] found that 12 weeks after IR, compared to control MSCs treatment resulted in a reduction in glandular follicular cell loss, cytoplasmic vacuolation, abnormal nuclei, and periductal and perivascular fibrosis. In addition, a study by Saylam et al. [43] compared the recovery of salivary gland microscopic morphology at different times. At 1 month after IR, periductal fibrosis and sclerosis of salivary gland ducts were significantly better after MSCs treatment compared with controls (*p* < 0.05). At 6 months after IR, edema, vacuolization, necrosis, ectasia, sclerosis, periductal fibrosis, and periductal sclerosis were better after MSCs treatment compared with controls (*p* < 0.05).

#### Macromorphology

Six studies [15, 30, 39–42] assessed macroscopic morphological improvement after treatment with MSCs. Three studies [15, 39, 40] assessed weight changes at 16 weeks after IR and found a significant increase in weight in the MSCs group compared to the control group. In addition, the study by An and Kim et al. [39, 40] also measured changes in salivary gland weight. It showed that gland weight was also significantly increased in the MSCs group compared to the control group. Three studies [30, 41, 42] assessed changes in body weight at 12 weeks after IR. The study by Shin et al. [41] showed a significant increase in body weight and gland weight in the MSCs treated mice compared to the control group. However, the other two studies [30, 42] showed an upward trend in body weight and salivary gland in the MSCs-treated mice/rats compared to the control group. But it lacked statistical significance.

### Radiation oral mucositis

#### Ulcer time

Two studies [14, 38] involved the exploration of ulcer latency times and the duration of ulcers. Maria et al. [38] showed that the latent duration of ulcers in control group was 9.3 ± 0.3 days. However, with using of MSCs, the latent duration of ulcers extended to 11.3 ± 0.9 days. Meanwhile, compared to the control group, the duration of ulceration was also reduced by 72% in the MSCs group. But, experiments by Schmidt et al. [14] showed that although the experimental group using MSCs could shorten the duration of ulcers compared to the control group, the ulcer incubation time was prolonged. And we can suppose that under the conditions of MSC transplantation, the regulation of RIOM in RT mouse models depends on the transplant time relative to the RT exposure time.

#### Micromorphology

Three studies [13, 35, 38] evaluated microscopic morphological improvements after treatment with MSCs. The study by Maria et al. [38] and the study by Elsaadany et al. [13] reported changes in epithelial height. And the mean values of epithelial height were significantly higher after MSCs treatment compared with the control group. In addition, the study by Elsaadany et al. [13] reported a significant decrease in the number of blood vessels, and better preservation of keratin and basal cell structure after MSCs treatment compared to controls after 7 days of irradiation [35].The study by Shen et al. [35] confirmed that, compared to control, after treatment with MSCs, radiation-induced loss of filopodiaulceration, disruption of the mucosal epithelial layer, and the degree of inflammatory cell infiltration were reduced. Furthermore, the mucosal thickness was better maintained.

#### Macromorphology

Two studies [35, 38] reported changes in ulcer size after treatment with MSCs. The results showed that the MSCs-treated group showed a smaller RIOM ulcer size compared to the control group at all time points tested after 15 consecutive days of observation. The study by Maria et al. [38] showed that the therapeutic benefit of MSCs depends on dose size and frequency, the number of doses, and the time of treatment initiation relative to the duration of RT exposure.

#### Other indicators

The study by Maria et al. [38] confirmed that MSCs treatment improved RIOM side effects. Compared to controls, the MSCs-treated animals had a significant improvement in hydration status, a significant reduction in weight loss, and a significant increase in the rate and extent of weight gain.

Elsaadany et al. [13] determined apoptosis, using the expression level of BCL-2 as an indicator for determination. The results showed that at 3 days and 7 days after IR, the expression level of BCL-2 was significantly increased in the group of the systemic use of stem cells compared to the normal control, and almost reached normal levels at 7 days after irradiation, with significant decrease in the apoptotic effect.

Schmidt et al. [14] studied the tolerance of the oral mucosa and expressed it as ED50 (the amount of radiation tested that would be expected to cause ulcers in 50% of the animals). The results showed that both split irradiation (3 Gy/day × 5 days) and single irradiation (15 Gy/dose) significantly increased the ED 50 value and significantly increased the residual tolerance of the oral mucosa in the MSCs treatment group compared to the normal control.

A study by Shen et al. [35] confirmed that mRNA expression of pro-inflammatory cytokines in the mucosa of mice after MSC treatment was significantly downregulated in MSCs. Also, MSCs enhanced the removal of ROS, reduced the production of radioactive ROS (*p* < 0.05), accelerated the recovery from mucositis, and protected tongue cells from cell death.

### Safety

The studies included in this review did not test the safety of MSCs for the treatment of radiation-induced xerostomia and oral mucositis in rodent models [13–15, 30, 35, 38–44].

## Discussion

This is the first systematic evaluation of the efficacy and safety of MSCs in radiation-induced oral complications. The results showed that MSCs exhibited good therapeutic efficacy in radiation-induced xerostomia and some improvement in radiation oral mucositis, despite differences in MSC source and graft type, the timing of administration, route of administration, dose, receptor type, and multiple models. Although previous reviews [45, 46] have explored the role of MSCs in oral dryness and oral mucositis, radiotherapy-induced xerostomia and oral mucositis have not been widely recognized, and whether MSCs have a therapeutic role in radiotherapy-induced oral dry mouth and oral mucositis and their therapeutic mechanisms have not been explored in detail. Therefore, we conducted a systematic evaluation to focus on this issue. Our review bridges the gap between previous experiments and confirms that the benefits of MSCs in radiation therapy-induced xerostomia and oral mucositis can ameliorate the discomfort they cause.

### Radiation-induced xerostomia

From the systematic evaluation of the eight included studies, it is evident that MSCs can demonstrate their therapeutic effects by increasing salivary protein secretion with SFR, shortening salivary retention time, anti-apoptosis, enhancing tissue cytoprotective effects, and effects on the outcomes of micro- and macro-morphological changes [15, 30, 39–44]. Paracrine action has long been one of the key mechanisms investigated for the ameliorative effects of MSCs on radiation-induced xerostomia.MSCs can prevent SFR decline and improve the symptoms of oral dryness through paracrine induction of anti-inflammatory and tissue regeneration genes (EGF, TGF-*α*,) [47]. Previous studies have shown that MSCs secrete TGFβ1 to regulate lymphocyte proliferation, differentiation, and survival to maintain tolerance and control the initiation and regression of the inflammatory response by regulating the chemotaxis, activation, and survival of lymphocytes, natural killer cells, dendritic cells, macrophages, mast cells, and granulocytes [48]. MSCs also secrete EGF, TGF-α, HGF, and VEGF to exert nutritional effects on salivary glandular vesicles, ducts, and mucosal epithelial cells, maintain normal salivary gland secretion and excretion [49], promote neovascularization, stimulate salivary gland cells proliferation, inhibit salivary gland cells apoptosis and improve the symptoms of oral dryness [50]. Also, paracrine-mediated immunomodulatory and trophic effects of stem cells may prevent the development of autoimmune activities by inhibiting immune cells from investigating tissue damage, establishing a regenerative microenvironment containing nutrient-active molecules, and promoting the proliferation of tissue cells and blood vessels [51] to restore damaged tissue and salivary organ functions after radiation injury [52]. Our included studies also showed that MSCs can paracrine EGF [40], FGF10 [41], HGF, and VEGF [44] leading to activation of PI3K and phosphorylation of downstream targets AKT and MDM2, inhibiting p53-mediated radiation-induced apoptotic pathway and protect salivary gland epithelial cells from radiation injury. Thus, MSCs promote the structural integrity of salivary glands in vitro and in vivo, and the preservation of endocrine function [41, 53, 54].

In addition, cellular transdifferentiation has been a controversial mechanism by which MSCs improve oral dryness. Previous studies have shown that MSCs can undergo a mesenchymal-to-epithelial transformation when induced by natural SG-specific extracellular matrix and then differentiate into corresponding salivary gland cells to repair damaged glands by replacing damaged salivary gland cells [55]. Furthermore, when MSCs are co-cultured with SGEC, MSCs can transdifferentiate into SGEC and promote amylase and mucopolysaccharide secretion from salivary gland epithelial cells by inducing salivary gland gene expression [45], promoting salivary secretion and alleviating oral dryness. However, recently Bhartiya et al. [23] made a strong generalization after reading and summarizing a large body of literature on the subject, suggesting that MSCs may not be able to differentiate into multiple adult cell types They are more like "paracrine providers" of tissue-resident stem cells. This is because similar beneficial effects are observed when their secretome (microvesicles or exosomes) are transplanted. Similarly, our included studies showed that only a minority of infused hAdMSCs transdifferentiated into salivary gland mast cells when treated with MSCs alone and produced amylase in vivo mainly through paracrine effects rather than transdifferentiation [30, 42]. This suggests that in the context of treatment with MSCs alone, MSCs may indirectly promote salivary cell survival, endogenous progenitor cell mobilization, or neointima formation. Ultimately, MSCs ameliorate radiation-induced xerostomia, mainly through paracrine effects rather than transdifferentiation [30, 42].

Although MSCs have therapeutic efficacy in radiation xerostomia, several factors/variables have been identified that may influence the outcomes of MSCs intervention. These factors mainly include the culture conditions and dependency vectors of MSC exosomes. The culture conditions of MSCs have been reported to affect their dryness and paracrine function and influence the therapeutic outcomes. An et al. [39] and Shin et al. [41] demonstrated that hypoxic conditions enhanced the paracrine activity of MSCs, the protein levels of secretions released by MSCs, and expressed higher levels of anti-apoptotic and angiogenic genes, including EGF, FGF10, HGF, IGF, and VEGF. A study by Mulyani et al. [44] also confirmed that hypoxic pretreatment could better induce the cellular repair process by better promoting the migration of MSCs in the ductal basement membrane and glandular vesicle cells. The delivery vehicle of MSCs is another factor to be considered and ideally tested. A study by Choi et al. [17] showed that delivery of MSCs as a vehicle in SIS MSCs not only maintained SG-specific cell expansion capacity but also organized glandular structures by enhancing glandular constituent cells (i.e., salivary epithelial cells, myoepithelial cells, and endothelial cells).

### Radiation-induced oral mucositis

We included four studies using MSCs in the treatment of radiation oral mucositis [13, 14, 35, 38] to assess the efficacy of MSCs in the treatment of radiation oral mucositis. Among the three components that we focused on assessing, two indicators (macroscopic and microscopic status of ulcers) were significantly improved and showed a positive effect of MSCs on RIOM (Table 4) [13, 14, 35, 38]. Although the current study failed to agree on the effect of MSCs on the improvement of ulcer latency and duration, previous studies confirmed that the latency and severity of ulcers due to RIOM correlate with the mouse strain chosen for the study [56] and the radiation dose [57]. This may be the reason why the effect of improvement in ulcer latency and duration in the included studies [14, 38] could not be agreed upon. We also observed that the concentration, mode of administration, and frequency of administration of MSCs in the included studies were not completely consistent. We can speculate that these factors also have an effect on ulcer latency and duration.

The current study found that MSCs are mainly involved in tissue mucosal repair in three ways. (1) Through direct differentiation [58], for example, bone marrow cells can be transplanted into the skin in response to wound stimulation, inducing the incorporation and differentiation of bone marrow-derived cells into non-hematopoietic skin structures and stimulating the regeneration of damaged skin tissue to promote skin wound healing. (2) Direct cellular interaction [59], such as direct interaction with cells of the innate and adaptive immune system, leading to the regulation of some effector functions. or regulating intracellular reactive oxygen species (ROS) levels by donating mitochondria to damaged cells, reducing the activation of ROS in damaged tissues, and ultimately improving the manifestation of tissue damage and inflammation by regulating cellular metabolism in damaged tissues. [60]. (3) Secretion of soluble factors [61], such as mesenchymal stem cells can secrete fibroblast growth factor [28], and one of its members, keratinocyte growth factor (KGF), can promote the proliferation of oral epithelial cells and enhance the radiation resistance of oral epithelial cells, thus improving radiation oral mucositis [62, 63].

Our included studies found that the paracrine effect of MSCs may be the most prominent mechanism for improving radiation oral mucositis. MSCs paracrine secrete anti-inflammatory and antioxidant cytokines and growth factors, which promote oral mucosal repair by promoting cell and tissue regeneration [13, 14, 35]. However, there is no definite conclusion on the specific cytokines secreted by MSCs. Further exploration is needed. As for the factors that promote cell and tissue regeneration, previous studies have suggested that the cytokines secreted by MSCs can promote mucosal repair by enhancing cell proliferation or inhibiting epithelial cell apoptosis, or a combination of both. For example, IL-11 secreted by MSCs can exert cytoprotective functions and reduce apoptosis by upregulating IL-11 receptor complex and heat shock protein 25 [32]. HGF secreted by MSCs induces proliferation of epithelial cells through phosphatidylinositol 3-kinase/Akt signaling pathway [33]. IGF-I secreted by MSCs can downregulate the expression of pro-apoptotic genes such as p53 or activate the expression of anti-apoptotic genes such as bc1-2 to inhibit radiation-induced apoptosis [34]. For anti-inflammatory factors, paracrine IL10 secreted by MSCs can down-regulate TNFα, INFβ, IL2, and other inflammatory factors to mediate the anti-inflammatory effects. In addition, some other unknown anti-inflammatory factors secreted by MSCs may promote anti-inflammatory effects by altering the inflammatory profile of Thelper1 cells toward a more anti-inflammatory Thelper2 profile and increasing the number of anti-inflammatory T regulatory cells [64, 65].

However, our included studies [13, 14, 35] also showed that the clonal characterization of stem cells in the tongue mucosa lining was identified by finding no clonal characterizations of transplanted stem cells or too few clonal characterizations of stem cells in the tongue mucosa lining, and the overall cell count in the oral mucosa was the same in the transplanted and irradiated groups. In addition, Shen et al. [35] explored the mechanism of action of MSCs in reducing cellular ROS levels, using transwell or direct co-culture systems of MSCs and tongue epithelial cells or fibroblasts to verify ROS levels, and found no significant difference in ROS levels between the two culture systems, which showed similar antioxidant effects. Therefore, these findings do not support for the time being the effects of MSCs on ameliorative effects of radiation oral mucositis were significantly correlated with transdifferentiation and direct cellular interaction.

Furthermore, we found that genetic modifications enhanced the role of MSCs in the treatment of radiation oral mucositis. Shen’s study [35] showed that MSCs-mediated CXCR2 overexpression by upregulating the expression of P-Akt and P-Erk1/2, which enhanced the migration ability and targeting ability of MSCs to the inflamed oral mucosa, prolonged the survival time and further improve the therapeutic effect of MSCs in radiation oral mucositis.

## Limitations

Although our conclusions suggest that MSCs are promising therapeutic agents, the use of MSCs for the treatment of radiation-induced oral complications is still in its infancy. There are still some critical steps to be taken to get through the FDA approval process and eventually into the clinic. First, all of the studies included in this evaluation were conducted on small animals. Therefore, future efforts should be made to move to larger animal models as well as multi-animal models. Second, future preclinical studies need to refine the pharmacological and toxicological studies of MSCs to elucidate in detail the pharmacological phenomena, mechanism of action (MOA), toxicity profile, toxic target organs, drug absorption, distribution, metabolism and excretion (ADME), and adverse effect profile of MSCs in radiation-induced xerostomia and oral mucositis. Third, we need to supplement the context of investigation new drug (IND) to refine and evaluate the clinical study protocol, investigator information, and provide detailed information on clinical procedures and the qualifications of the investigators. We also need to provide information on the production of MSCs, such as components, impurities, stabilities, source, and qualities,. To ensure that the company can adequately produce and supply stable batches of the drug. Fourth, we should follow the FDA guidelines and conduct clinical trials after IND approval, especially conducting large phase III clinical trials to fully validate the safety and efficacy to obtain a Biologics License Application (BLA).

## Conclusion

In this review, we comprehensively evaluated the efficacy and safety of MSCs for the treatment of radiation-induced xerostomia and oral mucositis based on available preclinical animal studies. Our study demonstrates that MSCs are effective in ameliorating radiation-induced xerostomia, and our study also suggests that MSCs may provide some minor therapeutic benefits for radiation-induced oral mucositis. In the future, we expect more researchers to carry out further evaluations of the effectiveness and safety of MSCs in improving radiation-induced xerostomia and oral mucositis to promote faster and better development of the stem cell field.

## Supplementary Information

**Additional file1**. Search Strategy.

## Data Availability

Not applicable.

## Abbreviations

SG: Salivary gland
RT: Radiation therapy
HNC: Head and neck cancer
IMRT: Intensity-modulated radiotherapy
RIOM: Radiation-induced oral mucositis
EV: Extracellular vesicle
SYRCLE: Systematic Review Centre for Laboratory Animal Experimentation
ROS: Reactive oxygen species
SFR: Salivary gland flow rate
KGF: Keratinocyte growth factor
EGF: Epidermal growth factor
MSC: Mesenchymal stem cell
AdMSC: Adipose tissue-derived mesenchymal stem cell
BM-MSC: Bone marrow-derived mesenchymal stem cell
PBS: Phosphate buffered saline
TUNEL: Terminal dUTP node end labeling
MOA: Mechanism of action
ADME: Absorption, distribution, metabolism and excretion
IND: Investigation new drug
BLA: Biologics License Application
FDA: Food and Drug Administration
