# Supplementary material for: Efficacy of mesenchymal stem cell therapy in rodent models of radiation-induced xerostomia and oral mucositis: a systematic review
Source: Stem Cell Res Ther. 2023 Apr 12;14:82. doi: 10.1186/s13287-023-03301-y (PMC10099931; doi:10.1186/s13287-023-03301-y)
Supplement: Supplementary file 1 — Additional file1. Search Strategy. [file 13287_2023_3301_MOESM1_ESM.docx]

**Supplementary 1. Search Strategy**

| **Electronic databases** | **Search** | **Search strategy** | **Results** |
| --- | --- | --- | --- |
| **PubMed** | #1 | (((((((Xerostomia) OR (Xerostomias)) OR (Hyposalivation)) OR (Hyposalivations)) OR (Asialia)) OR (Asialias)) OR ("Mouth Dryness")) OR ("Dryness, Mouth") | 21063 |
|  | #2 | (((((((Stomatitis) OR (Stomatitides)) OR ("Oral Mucositis")) OR ("Mucositides, Oral")) OR ("Oral Mucositides")) OR (Oromucositis)) OR (Oromucositides)) OR ("Mucositis, Oral") | 32778 |
|  | #3 | ((((((((((((((((((((((((((((((((((((((((((((((((((((((((((((((((((((((((((((((((((((((((((((((((((((((((((((((((((((((((((((((((Radiation) OR (Radiations)) OR ("Electromagnetic Waves")) OR ("Electromagnetic Wave")) OR ("Wave, Electromagnetic")) OR ("Waves, Electromagnetic")) OR ("Electromagnetic Energy")) OR ("Electromagnetic Energy")) OR ("Energies, Electromagnetic")) OR ("Energy, Electromagnetic")) OR ("Gamma Rays")) OR ("Gamma Ray")) OR ("Ray, Gamma")) OR ("Gamma Wave")) OR ("Gamma Waves")) OR ("Wave, Gamma")) OR ("Waves, Gamma")) OR ("X-Rays, Nuclear")) OR ("Nuclear X-Ray")) OR ("X Rays, Nuclear")) OR ("X-Ray, Nuclear")) OR ("Nuclear X-Rays")) OR ("Nuclear X Rays")) OR (Light)) OR (Photoradiation)) OR (Photoradiations)) OR ("Light, Visible")) OR ("Visible Light")) OR ("Radio Waves")) OR ("Radio Wave")) OR ("Wave, Radio")) OR ("Waves, Radio")) OR (Radiowaves)) OR (Radiowave)) OR ("Hertzian Waves")) OR ("Waves, Hertzian")) OR ("Very High Frequency Waves")) OR ("High Frequency Waves")) OR ("Frequency Wave, High")) OR ("Frequency Waves, High")) OR ("High Frequency Wave")) OR ("Wave, High Frequency")) OR ("Waves, High Frequency")) OR ("Short Waves")) OR ("Short Wave")) OR ("Wave, Short")) OR ("Waves, Short")) OR ("T-Rays")) OR ("T-Ray")) OR ("T Ray")) OR ("X-Rays")) OR ("X Rays")) OR ("X Ray")) OR ("Ray, X")) OR ("Rays, X")) OR ("Roentgen Rays")) OR ("Rays, Roentgen")) OR ("Xray")) OR ("Xrays")) OR ("Roentgen Ray")) OR ("Ray, Roentgen")) OR ("X-Ray")) OR ("Grenz Rays")) OR ("Rays, Grenz")) OR ("Grenz Ray")) OR ("Ray, Grenz")) OR ("Alpha Particles")) OR ("Alpha Particle")) OR ("Particle, Alpha")) OR ("Particles, Alpha")) OR ("Alpha Rays")) OR ("Alpha Ray")) OR ("Ray, Alpha")) OR ("Rays, Alpha")) OR ("Beta Particles")) OR ("Beta Particle")) OR ("Particle, Beta")) OR ("Particles, Beta")) OR ("Beta Rays")) OR ("Beta Ray")) OR ("Ray, Beta")) OR ("Rays, Beta")) OR ("HZE Particles")) OR ("HZE Particle")) OR ("Particle, HZE")) OR ("Particles, HZE")) OR ("Ultraviolet Rays")) OR ("Ray, Ultraviolet")) OR ("Ultraviolet Ray")) OR ("Ultra-Violet Rays")) OR ("Ray, Ultra-Violet")) OR ("Ultra Violet Rays")) OR ("Ultra-Violet Ray")) OR ("UV Light")) OR ("Light, UV")) OR ("Actinic Rays")) OR ("Actinic Ray")) OR ("Ray, Actinic")) OR ("Ultraviolet Light")) OR ("Light, Ultraviolet")) OR ("Black Light, Ultraviolet")) OR ("Ultraviolet Black Light")) OR ("Ultraviolet Black Lights")) OR (Radiotherapy)) OR (Radiotherapies)) OR ("Radiotherapy, Targeted")) OR ("Radiotherapies, Targeted")) OR ("Targeted Radiotherapies")) OR ("Targeted Radiotherapy")) OR (Sound)) OR (Sounds)) OR ("Sound Waves")) OR ("Sound Wave")) OR ("Wave, Sound")) OR ("Waves, Sound")) OR ("Elastic Waves")) OR ("Elastic Wave")) OR ("Wave, Elastic")) OR ("Waves, Elastic")) OR ("Acoustic Waves")) OR ("Acoustic Wave")) OR ("Wave, Acoustic")) OR ("Waves, Acoustic")) OR ("Solar Energy")) OR ("Energies, Solar")) OR ("Energy, Solar")) OR ("Solar Energies")) OR ("Solar Power")) OR ("Power, Solar") | 2,782,079 |
|  | #4 | (((((((((((((((((((((((((((((((((((((((((("Mesenchymal Stem Cells") OR ("Stem Cell, Mesenchymal")) OR ("Mesenchymal Stem Cell")) OR ("Stem Cells, Mesenchymal")) OR ("Bone Marrow Mesenchymal Stem Cells")) OR ("Bone Marrow Mesenchymal Stem Cell")) OR ("Bone Marrow Stromal Cells")) OR ("Bone Marrow Stromal Cell")) OR ("Bone Marrow Stromal Cells, Multipotent")) OR ("Multipotent Bone Marrow Stromal Cell")) OR ("Multipotent Bone Marrow Stromal Cells")) OR ("Adipose-Derived Mesenchymal Stem Cells")) OR ("Adipose Derived Mesenchymal Stem Cells")) OR ("Adipose-Derived Mesenchymal Stromal Cells")) OR ("Adipose Derived Mesenchymal Stromal Cells")) OR ("Mesenchymal Stem Cells, Adipose-Derived")) OR ("Mesenchymal Stem Cells, Adipose Derived")) OR ("Adipose-Derived Mesenchymal Stem Cell")) OR ("Adipose Derived Mesenchymal Stem Cell")) OR ("Adipose Tissue-Derived Mesenchymal Stem Cell")) OR ("Adipose Tissue Derived Mesenchymal Stem Cell")) OR ("Adipose Tissue-Derived Mesenchymal Stem Cells")) OR ("Adipose Tissue Derived Mesenchymal Stem Cells")) OR ("Adipose Tissue-Derived Mesenchymal Stromal Cells")) OR ("Adipose Tissue Derived Mesenchymal Stromal Cells")) OR ("Adipose Tissue-Derived Mesenchymal Stromal Cell")) OR ("Adipose Tissue Derived Mesenchymal Stromal Cell")) OR ("Mesenchymal Stromal Cells")) OR ("Mesenchymal Stromal Cell")) OR ("Stromal Cell, Mesenchymal")) OR ("Stromal Cells, Mesenchymal")) OR ("Multipotent Mesenchymal Stromal Cells")) OR ("Multipotent Mesenchymal Stromal Cell")) OR ("Mesenchymal Stromal Cells, Multipotent")) OR ("Mesenchymal Progenitor Cell")) OR ("Mesenchymal Progenitor Cells")) OR ("Progenitor Cell, Mesenchymal")) OR ("Progenitor Cells, Mesenchymal")) OR ("Wharton Jelly Cells")) OR ("Wharton's Jelly Cells")) OR ("Wharton's Jelly Cell")) OR ("Whartons Jelly Cells")) OR ("Bone Marrow Stromal Stem Cells") | 76,178 |
|  | #5 | (#1 OR #2 )AND #3 AND #4 | 32 |
| **Medline** | #1 | TS=(((((((Xerostomia) OR (Xerostomias)) OR (Hyposalivation)) OR (Hyposalivations)) OR (Asialia)) OR (Asialias)) OR ("Mouth Dryness")) OR ("Dryness, Mouth") | [8311](https://www.ncbi.nlm.nih.gov/pubmed/?cmd=HistorySearch&querykey=5) |
|  | #2 | TS=(((((((Stomatitis) OR (Stomatitides)) OR ("Oral Mucositis")) OR ("Mucositides, Oral")) OR ("Oral Mucositides")) OR (Oromucositis)) OR (Oromucositides)) OR ("Mucositis, Oral") | [27,075](https://www.ncbi.nlm.nih.gov/pubmed/?cmd=HistorySearch&querykey=6) |
|  | #3 | TS=((((((((((((((((((((((((((((((((((((((((((((((((((((((((((((((((((((((((((((((((((((((((((((((((((((((((((((((((((((((((((((((((Radiation) OR (Radiations)) OR ("Electromagnetic Waves")) OR ("Electromagnetic Wave")) OR ("Wave, Electromagnetic")) OR ("Waves, Electromagnetic")) OR ("Electromagnetic Energy")) OR ("Electromagnetic Energy")) OR ("Energies, Electromagnetic")) OR ("Energy, Electromagnetic")) OR ("Gamma Rays")) OR ("Gamma Ray")) OR ("Ray, Gamma")) OR ("Gamma Wave")) OR ("Gamma Waves")) OR ("Wave, Gamma")) OR ("Waves, Gamma")) OR ("X-Rays, Nuclear")) OR ("Nuclear X-Ray")) OR ("X Rays, Nuclear")) OR ("X-Ray, Nuclear")) OR ("Nuclear X-Rays")) OR ("Nuclear X Rays")) OR (Light)) OR (Photoradiation)) OR (Photoradiations)) OR ("Light, Visible")) OR ("Visible Light")) OR ("Radio Waves")) OR ("Radio Wave")) OR ("Wave, Radio")) OR ("Waves, Radio")) OR (Radiowaves)) OR (Radiowave)) OR ("Hertzian Waves")) OR ("Waves, Hertzian")) OR ("Very High Frequency Waves")) OR ("High Frequency Waves")) OR ("Frequency Wave, High")) OR ("Frequency Waves, High")) OR ("High Frequency Wave")) OR ("Wave, High Frequency")) OR ("Waves, High Frequency")) OR ("Short Waves")) OR ("Short Wave")) OR ("Wave, Short")) OR ("Waves, Short")) OR ("T-Rays")) OR ("T-Ray")) OR ("T Ray")) OR ("X-Rays")) OR ("X Rays")) OR ("X Ray")) OR ("Ray, X")) OR ("Rays, X")) OR ("Roentgen Rays")) OR ("Rays, Roentgen")) OR ("Xray")) OR ("Xrays")) OR ("Roentgen Ray")) OR ("Ray, Roentgen")) OR ("X-Ray")) OR ("Grenz Rays")) OR ("Rays, Grenz")) OR ("Grenz Ray")) OR ("Ray, Grenz")) OR ("Alpha Particles")) OR ("Alpha Particle")) OR ("Particle, Alpha")) OR ("Particles, Alpha")) OR ("Alpha Rays")) OR ("Alpha Ray")) OR ("Ray, Alpha")) OR ("Rays, Alpha")) OR ("Beta Particles")) OR ("Beta Particle")) OR ("Particle, Beta")) OR ("Particles, Beta")) OR ("Beta Rays")) OR ("Beta Ray")) OR ("Ray, Beta")) OR ("Rays, Beta")) OR ("HZE Particles")) OR ("HZE Particle")) OR ("Particle, HZE")) OR ("Particles, HZE")) OR ("Ultraviolet Rays")) OR ("Ray, Ultraviolet")) OR ("Ultraviolet Ray")) OR ("Ultra-Violet Rays")) OR ("Ray, Ultra-Violet")) OR ("Ultra Violet Rays")) OR ("Ultra-Violet Ray")) OR ("UV Light")) OR ("Light, UV")) OR ("Actinic Rays")) OR ("Actinic Ray")) OR ("Ray, Actinic")) OR ("Ultraviolet Light")) OR ("Light, Ultraviolet")) OR ("Black Light, Ultraviolet")) OR ("Ultraviolet Black Light")) OR ("Ultraviolet Black Lights")) OR (Radiotherapy)) OR (Radiotherapies)) OR ("Radiotherapy, Targeted")) OR ("Radiotherapies, Targeted")) OR ("Targeted Radiotherapies")) OR ("Targeted Radiotherapy")) OR (Sound)) OR (Sounds)) OR ("Sound Waves")) OR ("Sound Wave")) OR ("Wave, Sound")) OR ("Waves, Sound")) OR ("Elastic Waves")) OR ("Elastic Wave")) OR ("Wave, Elastic")) OR ("Waves, Elastic")) OR ("Acoustic Waves")) OR ("Acoustic Wave")) OR ("Wave, Acoustic")) OR ("Waves, Acoustic")) OR ("Solar Energy")) OR ("Energies, Solar")) OR ("Energy, Solar")) OR ("Solar Energies")) OR ("Solar Power")) OR ("Power, Solar") | [2,539,555](https://www.ncbi.nlm.nih.gov/pubmed/?cmd=HistorySearch&querykey=11) |
|  | #4 | TS=(((((((((((((((((((((((((((((((((((((((((("Mesenchymal Stem Cells") OR ("Stem Cell, Mesenchymal")) OR ("Mesenchymal Stem Cell")) OR ("Stem Cells, Mesenchymal")) OR ("Bone Marrow Mesenchymal Stem Cells")) OR ("Bone Marrow Mesenchymal Stem Cell")) OR ("Bone Marrow Stromal Cells")) OR ("Bone Marrow Stromal Cell")) OR ("Bone Marrow Stromal Cells, Multipotent")) OR ("Multipotent Bone Marrow Stromal Cell")) OR ("Multipotent Bone Marrow Stromal Cells")) OR ("Adipose-Derived Mesenchymal Stem Cells")) OR ("Adipose Derived Mesenchymal Stem Cells")) OR ("Adipose-Derived Mesenchymal Stromal Cells")) OR ("Adipose Derived Mesenchymal Stromal Cells")) OR ("Mesenchymal Stem Cells, Adipose-Derived")) OR ("Mesenchymal Stem Cells, Adipose Derived")) OR ("Adipose-Derived Mesenchymal Stem Cell")) OR ("Adipose Derived Mesenchymal Stem Cell")) OR ("Adipose Tissue-Derived Mesenchymal Stem Cell")) OR ("Adipose Tissue Derived Mesenchymal Stem Cell")) OR ("Adipose Tissue-Derived Mesenchymal Stem Cells")) OR ("Adipose Tissue Derived Mesenchymal Stem Cells")) OR ("Adipose Tissue-Derived Mesenchymal Stromal Cells")) OR ("Adipose Tissue Derived Mesenchymal Stromal Cells")) OR ("Adipose Tissue-Derived Mesenchymal Stromal Cell")) OR ("Adipose Tissue Derived Mesenchymal Stromal Cell")) OR ("Mesenchymal Stromal Cells")) OR ("Mesenchymal Stromal Cell")) OR ("Stromal Cell, Mesenchymal")) OR ("Stromal Cells, Mesenchymal")) OR ("Multipotent Mesenchymal Stromal Cells")) OR ("Multipotent Mesenchymal Stromal Cell")) OR ("Mesenchymal Stromal Cells, Multipotent")) OR ("Mesenchymal Progenitor Cell")) OR ("Mesenchymal Progenitor Cells")) OR ("Progenitor Cell, Mesenchymal")) OR ("Progenitor Cells, Mesenchymal")) OR ("Wharton Jelly Cells")) OR ("Wharton's Jelly Cells")) OR ("Wharton's Jelly Cell")) OR ("Whartons Jelly Cells")) OR ("Bone Marrow Stromal Stem Cells") | 76,089 |
|  | #5 | (#1 OR #2 )AND #3 AND #4 | 33 |
| **EMBASE** | #1 | (((((((Xerostomia) OR (Xerostomias)) OR (Hyposalivation)) OR (Hyposalivations)) OR (Asialia)) OR (Asialias)) OR ("Mouth Dryness")) OR ("Dryness, Mouth") | 44,237 |
|  | #2 | (((((((Stomatitis) OR (Stomatitides)) OR ("Oral Mucositis")) OR ("Mucositides, Oral")) OR ("Oral Mucositides")) OR (Oromucositis)) OR (Oromucositides)) OR ("Mucositis, Oral") | 53,501 |
|  | #3 | ((((((((((((((((((((((((((((((((((((((((((((((((((((((((((((((((((((((((((((((((((((((((((((((((((((((((((((((((((((((((((((((((Radiation) OR (Radiations)) OR ("Electromagnetic Waves")) OR ("Electromagnetic Wave")) OR ("Wave, Electromagnetic")) OR ("Waves, Electromagnetic")) OR ("Electromagnetic Energy")) OR ("Electromagnetic Energy")) OR ("Energies, Electromagnetic")) OR ("Energy, Electromagnetic")) OR ("Gamma Rays")) OR ("Gamma Ray")) OR ("Ray, Gamma")) OR ("Gamma Wave")) OR ("Gamma Waves")) OR ("Wave, Gamma")) OR ("Waves, Gamma")) OR ("X-Rays, Nuclear")) OR ("Nuclear X-Ray")) OR ("X Rays, Nuclear")) OR ("X-Ray, Nuclear")) OR ("Nuclear X-Rays")) OR ("Nuclear X Rays")) OR (Light)) OR (Photoradiation)) OR (Photoradiations)) OR ("Light, Visible")) OR ("Visible Light")) OR ("Radio Waves")) OR ("Radio Wave")) OR ("Wave, Radio")) OR ("Waves, Radio")) OR (Radiowaves)) OR (Radiowave)) OR ("Hertzian Waves")) OR ("Waves, Hertzian")) OR ("Very High Frequency Waves")) OR ("High Frequency Waves")) OR ("Frequency Wave, High")) OR ("Frequency Waves, High")) OR ("High Frequency Wave")) OR ("Wave, High Frequency")) OR ("Waves, High Frequency")) OR ("Short Waves")) OR ("Short Wave")) OR ("Wave, Short")) OR ("Waves, Short")) OR ("T-Rays")) OR ("T-Ray")) OR ("T Ray")) OR ("X-Rays")) OR ("X Rays")) OR ("X Ray")) OR ("Ray, X")) OR ("Rays, X")) OR ("Roentgen Rays")) OR ("Rays, Roentgen")) OR ("Xray")) OR ("Xrays")) OR ("Roentgen Ray")) OR ("Ray, Roentgen")) OR ("X-Ray")) OR ("Grenz Rays")) OR ("Rays, Grenz")) OR ("Grenz Ray")) OR ("Ray, Grenz")) OR ("Alpha Particles")) OR ("Alpha Particle")) OR ("Particle, Alpha")) OR ("Particles, Alpha")) OR ("Alpha Rays")) OR ("Alpha Ray")) OR ("Ray, Alpha")) OR ("Rays, Alpha")) OR ("Beta Particles")) OR ("Beta Particle")) OR ("Particle, Beta")) OR ("Particles, Beta")) OR ("Beta Rays")) OR ("Beta Ray")) OR ("Ray, Beta")) OR ("Rays, Beta")) OR ("HZE Particles")) OR ("HZE Particle")) OR ("Particle, HZE")) OR ("Particles, HZE")) OR ("Ultraviolet Rays")) OR ("Ray, Ultraviolet")) OR ("Ultraviolet Ray")) OR ("Ultra-Violet Rays")) OR ("Ray, Ultra-Violet")) OR ("Ultra Violet Rays")) OR ("Ultra-Violet Ray")) OR ("UV Light")) OR ("Light, UV")) OR ("Actinic Rays")) OR ("Actinic Ray")) OR ("Ray, Actinic")) OR ("Ultraviolet Light")) OR ("Light, Ultraviolet")) OR ("Black Light, Ultraviolet")) OR ("Ultraviolet Black Light")) OR ("Ultraviolet Black Lights")) OR (Radiotherapy)) OR (Radiotherapies)) OR ("Radiotherapy, Targeted")) OR ("Radiotherapies, Targeted")) OR ("Targeted Radiotherapies")) OR ("Targeted Radiotherapy")) OR (Sound)) OR (Sounds)) OR ("Sound Waves")) OR ("Sound Wave")) OR ("Wave, Sound")) OR ("Waves, Sound")) OR ("Elastic Waves")) OR ("Elastic Wave")) OR ("Wave, Elastic")) OR ("Waves, Elastic")) OR ("Acoustic Waves")) OR ("Acoustic Wave")) OR ("Wave, Acoustic")) OR ("Waves, Acoustic")) OR ("Solar Energy")) OR ("Energies, Solar")) OR ("Energy, Solar")) OR ("Solar Energies")) OR ("Solar Power")) OR ("Power, Solar") | 3,117,895 |
|  | #4 | (((((((((((((((((((((((((((((((((((((((((("Mesenchymal Stem Cells") OR ("Stem Cell, Mesenchymal")) OR ("Mesenchymal Stem Cell")) OR ("Stem Cells, Mesenchymal")) OR ("Bone Marrow Mesenchymal Stem Cells")) OR ("Bone Marrow Mesenchymal Stem Cell")) OR ("Bone Marrow Stromal Cells")) OR ("Bone Marrow Stromal Cell")) OR ("Bone Marrow Stromal Cells, Multipotent")) OR ("Multipotent Bone Marrow Stromal Cell")) OR ("Multipotent Bone Marrow Stromal Cells")) OR ("Adipose-Derived Mesenchymal Stem Cells")) OR ("Adipose Derived Mesenchymal Stem Cells")) OR ("Adipose-Derived Mesenchymal Stromal Cells")) OR ("Adipose Derived Mesenchymal Stromal Cells")) OR ("Mesenchymal Stem Cells, Adipose-Derived")) OR ("Mesenchymal Stem Cells, Adipose Derived")) OR ("Adipose-Derived Mesenchymal Stem Cell")) OR ("Adipose Derived Mesenchymal Stem Cell")) OR ("Adipose Tissue-Derived Mesenchymal Stem Cell")) OR ("Adipose Tissue Derived Mesenchymal Stem Cell")) OR ("Adipose Tissue-Derived Mesenchymal Stem Cells")) OR ("Adipose Tissue Derived Mesenchymal Stem Cells")) OR ("Adipose Tissue-Derived Mesenchymal Stromal Cells")) OR ("Adipose Tissue Derived Mesenchymal Stromal Cells")) OR ("Adipose Tissue-Derived Mesenchymal Stromal Cell")) OR ("Adipose Tissue Derived Mesenchymal Stromal Cell")) OR ("Mesenchymal Stromal Cells")) OR ("Mesenchymal Stromal Cell")) OR ("Stromal Cell, Mesenchymal")) OR ("Stromal Cells, Mesenchymal")) OR ("Multipotent Mesenchymal Stromal Cells")) OR ("Multipotent Mesenchymal Stromal Cell")) OR ("Mesenchymal Stromal Cells, Multipotent")) OR ("Mesenchymal Progenitor Cell")) OR ("Mesenchymal Progenitor Cells")) OR ("Progenitor Cell, Mesenchymal")) OR ("Progenitor Cells, Mesenchymal")) OR ("Wharton Jelly Cells")) OR ("Whartons Jelly Cells")) OR ("Whartons Jelly Cell")) OR ("Whartons Jelly Cells")) OR ("Bone Marrow Stromal Stem Cells") | 110,045 |
|  | #5 | (#1 OR #2 )AND #3 AND #4 | 68 |
| **Cochrane Library** | #1 | (((((((Xerostomia) OR (Xerostomias)) OR (Hyposalivation)) OR (Hyposalivations)) OR (Asialia)) OR (Asialias)) OR ("Mouth Dryness")) OR ("Dryness, Mouth"):ti,ab,kw | 5030 |
|  | #2 | (((((((Stomatitis) OR (Stomatitides)) OR ("Oral Mucositis")) OR ("Mucositides, Oral")) OR ("Oral Mucositides")) OR (Oromucositis)) OR (Oromucositides)) OR ("Mucositis, Oral"):ti,ab,kw | 5780 |
|  | #3 | ((((((((((((((((((((((((((((((((((((((((((((((((((((((((((((((((((((((((((((((((((((((((((((((((((((((((((((((((((((((((((((((((Radiation) OR (Radiations)) OR ("Electromagnetic Waves")) OR ("Electromagnetic Wave")) OR ("Wave, Electromagnetic")) OR ("Waves, Electromagnetic")) OR ("Electromagnetic Energy")) OR ("Electromagnetic Energy")) OR ("Energies, Electromagnetic")) OR ("Energy, Electromagnetic")) OR ("Gamma Rays")) OR ("Gamma Ray")) OR ("Ray, Gamma")) OR ("Gamma Wave")) OR ("Gamma Waves")) OR ("Wave, Gamma")) OR ("Waves, Gamma")) OR ("X-Rays, Nuclear")) OR ("Nuclear X-Ray")) OR ("X Rays, Nuclear")) OR ("X-Ray, Nuclear")) OR ("Nuclear X-Rays")) OR ("Nuclear X Rays")) OR (Light)) OR (Photoradiation)) OR (Photoradiations)) OR ("Light, Visible")) OR ("Visible Light")) OR ("Radio Waves")) OR ("Radio Wave")) OR ("Wave, Radio")) OR ("Waves, Radio")) OR (Radiowaves)) OR (Radiowave)) OR ("Hertzian Waves")) OR ("Waves, Hertzian")) OR ("Very High Frequency Waves")) OR ("High Frequency Waves")) OR ("Frequency Wave, High")) OR ("Frequency Waves, High")) OR ("High Frequency Wave")) OR ("Wave, High Frequency")) OR ("Waves, High Frequency")) OR ("Short Waves")) OR ("Short Wave")) OR ("Wave, Short")) OR ("Waves, Short")) OR ("T-Rays")) OR ("T-Ray")) OR ("T Ray")) OR ("X-Rays")) OR ("X Rays")) OR ("X Ray")) OR ("Ray, X")) OR ("Rays, X")) OR ("Roentgen Rays")) OR ("Rays, Roentgen")) OR ("Xray")) OR ("Xrays")) OR ("Roentgen Ray")) OR ("Ray, Roentgen")) OR ("X-Ray")) OR ("Grenz Rays")) OR ("Rays, Grenz")) OR ("Grenz Ray")) OR ("Ray, Grenz")) OR ("Alpha Particles")) OR ("Alpha Particle")) OR ("Particle, Alpha")) OR ("Particles, Alpha")) OR ("Alpha Rays")) OR ("Alpha Ray")) OR ("Ray, Alpha")) OR ("Rays, Alpha")) OR ("Beta Particles")) OR ("Beta Particle")) OR ("Particle, Beta")) OR ("Particles, Beta")) OR ("Beta Rays")) OR ("Beta Ray")) OR ("Ray, Beta")) OR ("Rays, Beta")) OR ("HZE Particles")) OR ("HZE Particle")) OR ("Particle, HZE")) OR ("Particles, HZE")) OR ("Ultraviolet Rays")) OR ("Ray, Ultraviolet")) OR ("Ultraviolet Ray")) OR ("Ultra-Violet Rays")) OR ("Ray, Ultra-Violet")) OR ("Ultra Violet Rays")) OR ("Ultra-Violet Ray")) OR ("UV Light")) OR ("Light, UV")) OR ("Actinic Rays")) OR ("Actinic Ray")) OR ("Ray, Actinic")) OR ("Ultraviolet Light")) OR ("Light, Ultraviolet")) OR ("Black Light, Ultraviolet")) OR ("Ultraviolet Black Light")) OR ("Ultraviolet Black Lights")) OR (Radiotherapy)) OR (Radiotherapies)) OR ("Radiotherapy, Targeted")) OR ("Radiotherapies, Targeted")) OR ("Targeted Radiotherapies")) OR ("Targeted Radiotherapy")) OR (Sound)) OR (Sounds)) OR ("Sound Waves")) OR ("Sound Wave")) OR ("Wave, Sound")) OR ("Waves, Sound")) OR ("Elastic Waves")) OR ("Elastic Wave")) OR ("Wave, Elastic")) OR ("Waves, Elastic")) OR ("Acoustic Waves")) OR ("Acoustic Wave")) OR ("Wave, Acoustic")) OR ("Waves, Acoustic")) OR ("Solar Energy")) OR ("Energies, Solar")) OR ("Energy, Solar")) OR ("Solar Energies")) OR ("Solar Power")) OR ("Power, Solar") | 100661 |
|  |  | (((((((((((((((((((((((((((((((((((((((((("Mesenchymal Stem Cells") OR ("Stem Cell, Mesenchymal")) OR ("Mesenchymal Stem Cell")) OR ("Stem Cells, Mesenchymal")) OR ("Bone Marrow Mesenchymal Stem Cells")) OR ("Bone Marrow Mesenchymal Stem Cell")) OR ("Bone Marrow Stromal Cells")) OR ("Bone Marrow Stromal Cell")) OR ("Bone Marrow Stromal Cells, Multipotent")) OR ("Multipotent Bone Marrow Stromal Cell")) OR ("Multipotent Bone Marrow Stromal Cells")) OR ("Adipose-Derived Mesenchymal Stem Cells")) OR ("Adipose Derived Mesenchymal Stem Cells")) OR ("Adipose-Derived Mesenchymal Stromal Cells")) OR ("Adipose Derived Mesenchymal Stromal Cells")) OR ("Mesenchymal Stem Cells, Adipose-Derived")) OR ("Mesenchymal Stem Cells, Adipose Derived")) OR ("Adipose-Derived Mesenchymal Stem Cell")) OR ("Adipose Derived Mesenchymal Stem Cell")) OR ("Adipose Tissue-Derived Mesenchymal Stem Cell")) OR ("Adipose Tissue Derived Mesenchymal Stem Cell")) OR ("Adipose Tissue-Derived Mesenchymal Stem Cells")) OR ("Adipose Tissue Derived Mesenchymal Stem Cells")) OR ("Adipose Tissue-Derived Mesenchymal Stromal Cells")) OR ("Adipose Tissue Derived Mesenchymal Stromal Cells")) OR ("Adipose Tissue-Derived Mesenchymal Stromal Cell")) OR ("Adipose Tissue Derived Mesenchymal Stromal Cell")) OR ("Mesenchymal Stromal Cells")) OR ("Mesenchymal Stromal Cell")) OR ("Stromal Cell, Mesenchymal")) OR ("Stromal Cells, Mesenchymal")) OR ("Multipotent Mesenchymal Stromal Cells")) OR ("Multipotent Mesenchymal Stromal Cell")) OR ("Mesenchymal Stromal Cells, Multipotent")) OR ("Mesenchymal Progenitor Cell")) OR ("Mesenchymal Progenitor Cells")) OR ("Progenitor Cell, Mesenchymal")) OR ("Progenitor Cells, Mesenchymal")) OR ("Wharton Jelly Cells")) OR ("Wharton's Jelly Cells")) OR ("Wharton's Jelly Cell")) OR ("Whartons Jelly Cells")) OR ("Bone Marrow Stromal Stem Cells") | 1879 |
|  |  | (#1 OR #2 )AND #3 AND #4 | 10 |
| **Web of Science** | #1 | TS=(((((((Xerostomia) OR (Xerostomias)) OR (Hyposalivation)) OR (Hyposalivations)) OR (Asialia)) OR (Asialias)) OR ("Mouth Dryness")) OR ("Dryness, Mouth") | 11,065 |
|  | #2 | TS= (((((((Stomatitis) OR (Stomatitides)) OR ("Oral Mucositis")) OR ("Mucositides, Oral")) OR ("Oral Mucositides")) OR (Oromucositis)) OR (Oromucositides)) OR ("Mucositis, Oral") | 37,110 |
|  | #3 | ((((((((((((((((((((((((((((((((((((((((((((((((((((((((((((((((((((((((((((((((((((((((((((((((((((((((((((((((((((((((((((((((Radiation) OR (Radiations)) OR ("Electromagnetic Waves")) OR ("Electromagnetic Wave")) OR ("Wave, Electromagnetic")) OR ("Waves, Electromagnetic")) OR ("Electromagnetic Energy")) OR ("Electromagnetic Energy")) OR ("Energies, Electromagnetic")) OR ("Energy, Electromagnetic")) OR ("Gamma Rays")) OR ("Gamma Ray")) OR ("Ray, Gamma")) OR ("Gamma Wave")) OR ("Gamma Waves")) OR ("Wave, Gamma")) OR ("Waves, Gamma")) OR ("X-Rays, Nuclear")) OR ("Nuclear X-Ray")) OR ("X Rays, Nuclear")) OR ("X-Ray, Nuclear")) OR ("Nuclear X-Rays")) OR ("Nuclear X Rays")) OR (Light)) OR (Photoradiation)) OR (Photoradiations)) OR ("Light, Visible")) OR ("Visible Light")) OR ("Radio Waves")) OR ("Radio Wave")) OR ("Wave, Radio")) OR ("Waves, Radio")) OR (Radiowaves)) OR (Radiowave)) OR ("Hertzian Waves")) OR ("Waves, Hertzian")) OR ("Very High Frequency Waves")) OR ("High Frequency Waves")) OR ("Frequency Wave, High")) OR ("Frequency Waves, High")) OR ("High Frequency Wave")) OR ("Wave, High Frequency")) OR ("Waves, High Frequency")) OR ("Short Waves")) OR ("Short Wave")) OR ("Wave, Short")) OR ("Waves, Short")) OR ("T-Rays")) OR ("T-Ray")) OR ("T Ray")) OR ("X-Rays")) OR ("X Rays")) OR ("X Ray")) OR ("Ray, X")) OR ("Rays, X")) OR ("Roentgen Rays")) OR ("Rays, Roentgen")) OR ("Xray")) OR ("Xrays")) OR ("Roentgen Ray")) OR ("Ray, Roentgen")) OR ("X-Ray")) OR ("Grenz Rays")) OR ("Rays, Grenz")) OR ("Grenz Ray")) OR ("Ray, Grenz")) OR ("Alpha Particles")) OR ("Alpha Particle")) OR ("Particle, Alpha")) OR ("Particles, Alpha")) OR ("Alpha Rays")) OR ("Alpha Ray")) OR ("Ray, Alpha")) OR ("Rays, Alpha")) OR ("Beta Particles")) OR ("Beta Particle")) OR ("Particle, Beta")) OR ("Particles, Beta")) OR ("Beta Rays")) OR ("Beta Ray")) OR ("Ray, Beta")) OR ("Rays, Beta")) OR ("HZE Particles")) OR ("HZE Particle")) OR ("Particle, HZE")) OR ("Particles, HZE")) OR ("Ultraviolet Rays")) OR ("Ray, Ultraviolet")) OR ("Ultraviolet Ray")) OR ("Ultra-Violet Rays")) OR ("Ray, Ultra-Violet")) OR ("Ultra Violet Rays")) OR ("Ultra-Violet Ray")) OR ("UV Light")) OR ("Light, UV")) OR ("Actinic Rays")) OR ("Actinic Ray")) OR ("Ray, Actinic")) OR ("Ultraviolet Light")) OR ("Light, Ultraviolet")) OR ("Black Light, Ultraviolet")) OR ("Ultraviolet Black Light")) OR ("Ultraviolet Black Lights")) OR (Radiotherapy)) OR (Radiotherapies)) OR ("Radiotherapy, Targeted")) OR ("Radiotherapies, Targeted")) OR ("Targeted Radiotherapies")) OR ("Targeted Radiotherapy")) OR (Sound)) OR (Sounds)) OR ("Sound Waves")) OR ("Sound Wave")) OR ("Wave, Sound")) OR ("Waves, Sound")) OR ("Elastic Waves")) OR ("Elastic Wave")) OR ("Wave, Elastic")) OR ("Waves, Elastic")) OR ("Acoustic Waves")) OR ("Acoustic Wave")) OR ("Wave, Acoustic")) OR ("Waves, Acoustic")) OR ("Solar Energy")) OR ("Energies, Solar")) OR ("Energy, Solar")) OR ("Solar Energies")) OR ("Solar Power")) OR ("Power, Solar") | 7,849,554 |
|  | #4 | (((((((((((((((((((((((((((((((((((((((((("Mesenchymal Stem Cells") OR ("Stem Cell, Mesenchymal")) OR ("Mesenchymal Stem Cell")) OR ("Stem Cells, Mesenchymal")) OR ("Bone Marrow Mesenchymal Stem Cells")) OR ("Bone Marrow Mesenchymal Stem Cell")) OR ("Bone Marrow Stromal Cells")) OR ("Bone Marrow Stromal Cell")) OR ("Bone Marrow Stromal Cells, Multipotent")) OR ("Multipotent Bone Marrow Stromal Cell")) OR ("Multipotent Bone Marrow Stromal Cells")) OR ("Adipose-Derived Mesenchymal Stem Cells")) OR ("Adipose Derived Mesenchymal Stem Cells")) OR ("Adipose-Derived Mesenchymal Stromal Cells")) OR ("Adipose Derived Mesenchymal Stromal Cells")) OR ("Mesenchymal Stem Cells, Adipose-Derived")) OR ("Mesenchymal Stem Cells, Adipose Derived")) OR ("Adipose-Derived Mesenchymal Stem Cell")) OR ("Adipose Derived Mesenchymal Stem Cell")) OR ("Adipose Tissue-Derived Mesenchymal Stem Cell")) OR ("Adipose Tissue Derived Mesenchymal Stem Cell")) OR ("Adipose Tissue-Derived Mesenchymal Stem Cells")) OR ("Adipose Tissue Derived Mesenchymal Stem Cells")) OR ("Adipose Tissue-Derived Mesenchymal Stromal Cells")) OR ("Adipose Tissue Derived Mesenchymal Stromal Cells")) OR ("Adipose Tissue-Derived Mesenchymal Stromal Cell")) OR ("Adipose Tissue Derived Mesenchymal Stromal Cell")) OR ("Mesenchymal Stromal Cells")) OR ("Mesenchymal Stromal Cell")) OR ("Stromal Cell, Mesenchymal")) OR ("Stromal Cells, Mesenchymal")) OR ("Multipotent Mesenchymal Stromal Cells")) OR ("Multipotent Mesenchymal Stromal Cell")) OR ("Mesenchymal Stromal Cells, Multipotent")) OR ("Mesenchymal Progenitor Cell")) OR ("Mesenchymal Progenitor Cells")) OR ("Progenitor Cell, Mesenchymal")) OR ("Progenitor Cells, Mesenchymal")) OR ("Wharton Jelly Cells")) OR ("Wharton's Jelly Cells")) OR ("Wharton's Jelly Cell")) OR ("Whartons Jelly Cells")) OR ("Bone Marrow Stromal Stem Cells") | 126,501 |
|  | #5 | (#1 OR #2 )AND #3 AND #4 | 57 |
